# Biomarker Validation of Dietary Intervention in Two Multiethnic Populations

**Published:** 2006-03-15

**Authors:** A. Heather Eliassen, Graham A Colditz, Karen E Peterson, Jeremy D Furtado, Martha E Fay, Glorian Sorensen, Karen M Emmons

**Affiliations:** Channing Laboratory, Department of Medicine, Brigham and Women’s Hospital and Harvard Medical School; Dr Eliassen is also affiliated with the Department of Epidemiology, Harvard School of Public Health, Boston, Mass; Channing Laboratory, Department of Medicine, Brigham and Women’s Hospital and Harvard Medical School, Boston, Mass, and Department of Epidemiology, Harvard School of Public Health, Boston, Mass; Department of Society, Human Development, and Health and Department of Nutrition, Harvard School of Public Health, Boston, Mass; Department of Nutrition, Harvard School of Public Health, Boston, Mass; Department of Environmental Health, Harvard School of Public Health, Boston, Mass; Department of Society, Human Development, and Health, Harvard School of Public Health, Boston, Mass, and Center for Community-Based Research, Dana–Farber Cancer Institute, Boston, Mass; Department of Society, Human Development, and Health, Harvard School of Public Health, Boston, Mass, and Center for Community-Based Research, Dana–Farber Cancer Institute, Boston, Mass

## Abstract

**Introduction:**

Intervention studies have been designed to change dietary and lifestyle factors associated with chronic diseases, but self-reported behavior change may incorporate intervention-related bias. This study examines plasma nutrient concentration and correlations with self-reports in the Healthy Directions intervention study. The Healthy Directions intervention studies were designed to increase multivitamin use, fruit and vegetable consumption, and physical activity in working-class, multiethnic populations.

**Methods:**

Participants in both studies completed interviewer-administered questionnaires that collected information on sociodemographic and health behavior characteristics. Postintervention blood samples were collected from 209 participants and pooled in pairs within study and within intervention group.

**Results:**

We found significantly higher plasma concentrations of retinol (*P* = .01) and α-carotene (*P* = .03) in the intervention than in the usual care group. Self-reported multivitamin users had significantly higher concentrations of retinol (*P* < .001), β-carotene (*P* = .02), and α-tocopherol (*P* < .001). Those who reported four or more fruit and vegetable servings per day had higher lutein and zeaxanthin (*P* = .05) and β-cryptoxanthin (*P* = .05) concentrations than those consuming fewer. Plasma nutrient concentrations were associated with reported multivitamin use and fruit and vegetable intake, but the correlations were generally higher in the usual care group.

**Conclusion:**

We found significant postintervention differences in plasma carotenoid and tocopherol concentrations by treatment group, multivitamin use, and fruit and vegetable intake. However, because we only obtained postintervention blood samples, we were unable to assess preintervention-to-postintervention changes in plasma nutrients. Self-reported intakes were significantly correlated with plasma nutrient concentrations, but the strength of the correlations differed by group, suggesting some intervention-related bias in the questionnaire responses.

## Introduction

Epidemiologic evidence supports an association between diet and several chronic diseases, including cardiovascular disease, type 2 diabetes, and several types of cancer (1-6). Intervention trials designed to promote dietary change are important to determine effective ways to implement change in populations with disparate risk behaviors and outcomes. The Healthy Directions studies within the Harvard Cancer Prevention Program Project (HCPPP) have implemented interventions through worksites and health care centers, focusing on a multiethnic, working-class (e.g., clerical, sales, skilled and unskilled labor) population (7-9). The aims of the interventions were to decrease red meat intake and increase fruit and vegetable intake, multivitamin use, and physical activity.

Differential overreporting is a potential problem when monitoring change in intervention studies. People who have been advised to change their diet may have an increased awareness of their intake and a desire to seem compliant (10,11). Self-reported change at the end of follow-up may reflect intervention-related bias rather than actual changes in diet. Therefore, validation studies are an important component of dietary intervention studies (12,13). An ideal validation study of a diet-related intervention would accomplish three goals: validate that the questionnaire is adequately measuring intake, validate the questionnaire-based dietary change from preintervention to postintervention, and finally, calibrate the primary data collection tool to eliminate potential intervention-related biases. These goals are often difficult to accomplish, particularly with budget restrictions common in many intervention studies.

To address these goals within the Healthy Directions studies, we conducted an ancillary study to examine plasma nutrient levels within a subset of participants at the end of follow-up. We present the results of these analyses, along with a discussion of the benefits and limitations of conducting such a study and suggestions for incorporating biomarker validation in future intervention studies.

## Methods

### Population

Healthy Directions encompasses two intervention studies in the greater Boston, Mass, metropolitan area — Healthy Directions–Health Centers (HC) and Healthy Directions–Small Businesses (SB) — which were both designed to increase fruit and vegetable consumption, multivitamin use, and physical activity and decrease red meat consumption. Randomization was performed by organization, with all members of a health center or small business randomized to either the intervention or the usual care group (May 2000–January 2002). The final assessment was completed by 1954 participants in the HC study and 1408 participants in the SB study (January 2001–November 2002). Details of these studies have been published elsewhere (7-9). The Healthy Directions studies were approved by the institutional review board at the Dana-Farber Cancer Institute.

At the final assessment, a subset of Healthy Directions participants was randomly selected and invited into our ancillary study, with the goal of recruiting 200 participants. Of 672 participants who were invited, 214 people donated blood samples from July 2001 through July 2002 (HC) and May through September 2002 (SB); 113 were ineligible, 205 declined to participate, and 140 did not participate for other reasons. Sociodemographic and health behavior characteristics did not differ substantially between those who were invited and gave a blood sample and those who were invited but did not participate. The blood sample donors had a mean age of 47.7 years, a mean body mass index (BMI) of 27.8 kg/m^2^, and a mean fruit and vegetable consumption of 3.5 servings per day; 49% were female, 45% were regular multivitamin users, 69% were white, 57% had some education after high school, and 45% had an income of less than $50,000 per year. Those who did not participate had a mean age of 48.0 years, a mean BMI of 27.1 kg/m^2^, and a mean fruit and vegetable consumption of 3.3 servings per day; 52% were female, 46% were regular multivitamin users, 61% were white, 56% had some education after high school, and 49% had an income of less than $50,000. Of the 209 samples analyzed, 94 were from the usual care group and 115 from the intervention group; 5 samples appeared hemolyzed and were excluded.

### Sociodemographic and dietary assessment

Participants in both studies completed interviewer-administered questionnaires that collected information on sociodemographic and health behavior characteristics, including date of birth, sex, level of education completed, household income, racial and ethnic background, smoking status, height, weight, and physical activity.

Fruit and vegetable consumption was measured using a survey developed for the National Cancer Institute's 5-A-Day for Better Health research projects (14); details of the use of this survey in the Healthy Directions studies have been published elsewhere (7-9). Usual consumption during the last 4 weeks of seven common foods and beverages (orange and grapefruit juice, other 100% fruit juice, green salad, fried potatoes, white potatoes other than fried, fruit, and other vegetables) was assessed; frequency categories ranged from never to five or more times per day. Responses were recoded to equivalent servings per day. Participants were also asked to report the frequency of multivitamin use in days per week.

### Laboratory analyses

Blood samples were collected at the worksite by a trained phlebotomist or at the health center by laboratory personnel and transported to Dana-Farber Cancer Institute for processing; samples were stored at −80° C until analysis. Blood samples were pooled in pairs within study (HC or SB) and treatment group (usual care or intervention) to reduce costs (12,15). Plasma carotenoids (retinol, α-carotene, *cis-*β-carotene, *trans-*β-carotene, total β-carotene, *cis-*lycopene, *trans-*lycopene, total lycopene, lutein and zeaxanthin combined, and β-cryptoxanthin) and tocopherols (α-tocopherol, δ-tocopherol, and γ-tocopherol) were measured by the Harvard School of Public Health Vitamin Analysis Laboratory using high-performance liquid chromatography (16). Internal laboratory quality control analyses yielded within-batch coefficients of variation ranging from 3.3% (α-carotene) to 5.4% (lutein and zeaxanthin).

### Statistical analyses

Means and proportions of study population characteristics were calculated by treatment group within the whole ancillary study and within the HC and SB subgroups. Nutrient analyses were conducted by pairs, not individually. Means of plasma nutrient concentrations were calculated by treatment group, reported fruit and vegetable intake (<4 or ≥4 servings per day), and reported multivitamin use (<6 or ≥6 days per week); *t* tests were used to test the difference between means of two samples, assuming unequal variances. Analyses by fruit and vegetable intake and multivitamin use were restricted to pairs concordant on the stratification factor. For example, only pairs in which both individuals were regular multivitamin users or both were nonusers were included, whereas pairs in which one individual was a regular user and the other was not were excluded. Although the goal of the study was to increase fruit and vegetable intake to 5 or more servings per day, it was not feasible to stratify at this level because only three pairs were identified in which both individuals consumed 5 or more servings per day. Spearman correlation coefficients were calculated between the pairs' plasma nutrients and the pair-averaged reported fruit and vegetable intake (0–9 servings per day) and multivitamin use (0–7 days per week). Results were considered statistically significant if *P* < .05. All analyses were conducted using SAS software, version 8 (SAS Institute, Inc, Cary, NC).

## Results

Characteristics of the study population at blood collection by treatment group (within each study and within the whole ancillary study) are presented in Table 1. The proportion of women varied between the studies but was similar between groups when the studies were combined (46% in the usual care group and 51% in the intervention group). The usual care group in the HC study had a higher prevalence of black participants (44%) than the other groups, which had more similar racial distributions. Higher income was more prevalent in the HC study intervention group, and the HC study had a higher prevalence of college-educated participants than the SB study. The distribution of smoking was similar between intervention groups in each study, but current smoking was more prevalent in the SB study. The HC intervention group was older (54 years) than the usual care group (48 years), and the HC study participants were slightly older and had a higher BMI than the SB participants. At the end of follow-up, fruit and vegetable consumption was higher in the intervention group, not only in each study but also when studies were combined (3.8 servings per day for the intervention group and 3.1 servings per day for the usual care group). Similarly, when studies were combined, the prevalence of multivitamin use was higher in the intervention group (57%) than the usual care group (30%).

Concentrations of several nutrients differed between the usual care and intervention groups, combined across studies (Table 2). Significantly higher concentrations of retinol (*P* = .01) and α-carotene (*P* = .03) were detected in the intervention group than the usual care group, which in turn had higher levels of δ-tocopherol (*P* = .02) and γ-tocopherol (*P* = .002). 

When participants were stratified by reported multivitamin use (concordant pairs), those who used multivitamins 6 or more days per week had significantly higher concentrations of retinol (*P* < .001), *cis-*β-carotene (*P* = .01), *trans-*β-carotene (*P* = .02), total β-carotene (*P* = .02), and α-tocopherol (*P* < .001) (Table 3). γ-Tocopherol was significantly higher in those who were not regular multivitamin users (*P* = .002). When stratified by reported fruit and vegetable intake (concordant pairs), participants who consumed 4 or more servings of fruits and vegetables per day had higher concentrations of lutein and zeaxanthin (*P* = .05) and β-cryptoxanthin (*P* = .05) than those who ate fewer than 4 servings (Table 4).

Correlations between plasma nutrients and reported multivitamin intake varied by treatment group (Table 5). Retinol was correlated with multivitamin use overall (*r* = 0.36, *P* < .001) but was not significant in the usual care group (*P* = .08). β-Carotene was more strongly and significantly correlated with intake in the usual care group (*r* = 0.39, *P* = .01 for *cis*-β-carotene; *r* = 0.34, *P* = .02 for *trans*-β-carotene; and *r* = 0.33, *P* = .02 for total β-carotene). α-Tocopherol was significantly correlated with multivitamin use in both groups (usual care: *r* = 0.35, *P* = .01; intervention: *r* = 0.54, *P* < .001). γ-Tocopherol was significantly inversely associated with multivitamin use in both groups (usual care: *r* = –0.38, *P* = .01; intervention: *r* = –0.40, *P* = .002). 

Correlations between plasma nutrients and reported fruit and vegetable intake also differed by treatment group (Table 6). Fruit and vegetable intake was significantly directly correlated with α-carotene when both groups were combined, (*r* = 0.22, *P* = .02) and with *cis-*β-carotene (*r* = 0.34, *P* = .02), *trans-*β-carotene (*r* = 0.39, *P* = .006), and total β-carotene (*r* = 0.39, *P* = .006) in the usual care group but not in the intervention group (*r* = 0.14–0.17, *P* = .21–.28). In the usual care group, the correlations of lutein and zeaxanthin (*r* = 0.31, *P* = .03) and β-cryptoxanthin (*r* = 0.35, *P* = .01) with fruit and vegetable intake were higher than in the intervention group (both *r* = 0.23, *P* = .08, .09).

## Discussion

To confirm the questionnaire-based findings of dietary change, we examined plasma nutrient concentrations at the end of follow-up by treatment status and found higher levels of retinol and α-carotene in the treatment group. To further explore nutrient differences, we stratified the pairs by multivitamin use and fruit and vegetable consumption instead of treatment group. We found higher retinol, β-carotene, and α-Tocopherol in participants who reported regular multivitamin use than in those who used multivitamins infrequently or not at all and higher lutein and zeaxanthin and β-cryptoxanthin in participants who reported consuming 4 or more servings of fruits and vegetables per day than in those who ate fewer. To verify the data collected by questionnaire, we examined correlations between reported multivitamin use and fruit and vegetable consumption. Multivitamin use was significantly directly correlated with retinol, *cis-*β-carotene, *trans-*β-carotene, total β-carotene, and α-Tocopherol and significantly inversely associated with γ-Tocopherol. However, the β-carotene components were only significantly correlated with multivitamin use in the usual care group; retinol and γ-Tocopherol were more highly correlated in the intervention group. Correlations between reported fruit and vegetable intake and *cis-*β-carotene, *trans-*β-carotene, total β-carotene, lutein and zeaxanthin, and β-cryptoxanthin were only significant in the usual care group; α-carotene was significantly correlated with consumption when both groups were combined. We did not have adequate data to calibrate the survey responses to the measured plasma nutrient levels.

The nutrient differences and correlations by multivitamin use and fruit and vegetable intake were expected. Higher plasma α-carotene, β-carotene, and α-Tocopherol levels have been observed after multivitamin supplementation (17-20). Common multivitamin formulations often contain retinol, β-carotene, and α-Tocopherol but are less likely to contain zeaxanthin or β-cryptoxanthin (21). The significantly higher levels of δ- and γ-Tocopherol among nonusers is likely a result of supplementation with γ-Tocopherol (the most common tocopherol in multivitamins), because it reduces plasma concentrations of δ- and γ-Tocopherol (22). Plasma carotenoid levels reflect consumption of carotenoid-rich foods (23-25) and have been shown to be an effective measure of a dietary intervention (26,27). Participants who reported higher fruit and vegetable intakes had higher plasma concentrations of lutein and zeaxanthin (which are commonly found in dark green, leafy vegetables) and β-cryptoxanthin (which is found in citrus fruit and orange juice) (28).

The higher concentrations of some plasma nutrients at the end of follow-up in the intervention group support the findings of significant increases in reported fruit and vegetable consumption and multivitamin use in these groups in the HC and SB studies (29,30). However, given that some characteristics were not well-balanced between the treatment groups among the subset of participants in the ancillary blood study, the nutrient differences may have been influenced by factors unrelated to the intervention. For instance, the higher prevalence of more highly educated, higher income participants in the intervention group may have contributed to the differing nutrition status; for example, they may have had a higher intake of fruits and vegetables at baseline and follow-up but not as a result of the intervention. In addition, the intervention group also had a higher mean age and BMI and slightly higher level of physical activity. Older age has been associated with higher concentrations of retinol and carotenoids (31,32). Although micronutrient concentrations are less likely than macronutrients to be affected by caloric intake, it is still possible that people who consume more calories (e.g., those with higher BMIs, higher physical activity levels, or both) also consume more micronutrients and thus may have higher plasma concentrations of micronutrients (12). Given that blood samples were pooled only accounting for treatment group and analyzed in pairs, we were unable to adjust our results for these factors.

Pooling the blood samples also decreased our sample size when stratifying by multivitamin use and fruit and vegetable intake, because we restricted the analyses to pairs that were concordant with respect to the dichotomized stratification factors. Despite the decreased sample size, we were still able to detect differences between the concordant pairs. However, although differences in nutrient concentrations were found by reported fruit, vegetable, and multivitamin intake, it is difficult to determine whether these differences are the result of the intervention, because only postintervention samples were collected. If preintervention and postintervention samples were available, within-person changes in plasma nutrient concentrations could have been compared with changes in reported intake.

Overall correlations of reported intake of multivitamins and fruits and vegetables with plasma nutrients suggest that the 5-A-Day survey adequately discriminated between individuals. However, the difference in correlations between the intervention and usual care groups is a concern. The nonsignificant correlations in the intervention group suggest that the questionnaire is not adequately measuring intake in this group. Although it is possible that biomarkers are representative of longer-term intake and may not reflect dietary changes within 1 year, dietary supplement studies suggest that increases in plasma concentrations of retinol, carotenoids, and tocopherols are apparent within 4 to 16 weeks of supplementation (33,34). Perhaps a more plausible source of this error is that participants who did not change their eating behaviors as advised overreported their fruit and vegetable consumption on the follow-up survey. Thus, it is possible that the questionnaires incorporate a systematic bias in the intervention group. Similar results have been found in other intervention studies. For instance, in a dietary intervention study, the ratio of reported fiber intake to fecal fiber content increased from baseline to 12 months, suggesting that fiber intake was overreported at the end of follow-up (35). Similarly, systematic underreporting of caloric intake in intervention groups was reported in a study of childhood obesity (11). Thus, when participants strive to comply with difficult interventions such as dietary changes, systematic bias may affect the results.

The results of this ancillary blood study suggest that the behavioral change intervention altered intake of multivitamins, fruits, and vegetables. However, several limitations to this validation study prompt a few recommendations for future studies. First, given the potential for intervention-related bias, data could be collected to calibrate or correct survey responses to minimize the impact of the bias. For example, a third instrument not prone to systematic, intervention-related bias could be used to regress intake on the nutrient biomarkers to calculate a calibration factor that could be applied to the survey data (12).

Second, cost-reduction measures should be planned to maintain a breadth of analysis options in the validation study. Blood samples were pooled to reduce assay costs so that we could conduct an ancillary study within the budget. Pooling samples is an effective way to perform a preliminary screen by comparing means among groups or to develop more specific hypotheses (15). However, pairing prevented us from adjusting for individual characteristics and reduced our effective sample size for some analyses. If cost reduction is necessary, pooling could be accomplished with carefully matched pairs so that the individuals in a set would be more similar with respect to potential confounders or with respect to nutrient intake (e.g., by deciles of reported fruit and vegetable intake). Thus, samples should be collected and stored individually, allowing time to match individuals on factors collected over the course of the study, and pooled just before being sent to the laboratory for analysis. Alternatively, other measures might be taken to reduce costs in future studies, such as minimizing the number of assays. For instance, a few biomarkers could be chosen to reflect hallmark components of multivitamins, fruits, and vegetables, such as red blood cell folate and plasma α-Tocopherol, β-carotene, and lutein and zeaxanthin (12,17-20,23-25), instead of a more extensive panel.

Finally, given the difficulty of validating change in reported intake with only postintervention biomarkers, preintervention and postintervention blood samples could be used to compare biomarker changes within individuals, thus removing the variability caused by individual metabolic differences (12). For example, change in serum cholesterol concentration is a relatively good marker of change in saturated fat intake, whereas a cross-sectional measure of cholesterol is likely more closely related to individual metabolic differences than to saturated fat intake, given the strict homeostatic regulation within individuals (12,36,37). Thus, improvements could be made to produce more valuable data in future validation studies.

Overall, the ancillary blood study was an important component of the Healthy Directions behavior intervention studies. We achieved the first goal of a validation study in that we found correlations between plasma concentrations of several nutrients and reported intake of fruits, vegetables, and multivitamins. However, the correlation analysis also revealed potential intervention-related bias. We found group differences in the postintervention samples, but other factors may have contributed to the differences in nutrient concentrations. Without preintervention blood samples, we could not compare the effect of intervention measured by reported change with change in plasma nutrients. To incorporate a more thorough validation component, future intervention studies might include preintervention and postintervention blood samples, with fewer assays or pooling that accounts for more matching factors to save costs.

## Figures and Tables

**Table 1:** Characteristics of Healthy Directions Ancillary Blood Study Participants, by Study and Treatment Group

**Characteristic**	**Health Center (HC) Participants**		**Small Business (SB) Participants**		**HC and SB Participants Combined**	

	**Usual Care n = 41**	**Intervention n = 54**	**Usual Care n = 53**	**Intervention n = 61**	**Usual Care n = 94**	**Intervention n = 115**

**Sex, %**						

Male	27	41	75	56	54	49
Female	73	59	25	44	46	51

**Race, %**						

Asian or Pacific Islander	0	0	6	0	3	0
Black	44	15	0	0	19	7
Hispanic	12	6	11	15	12	10
Mixed or other	5	4	8	7	6	5
White	39	76	75	79	60	77

**Annual income, %**						

<$20,000	10	8	2	9	5	9
$20,000-$49,999	44	26	46	36	45	31
≥$50,000	46	66	52	55	49	60

**Education, %**						

High school or less	33	37	45	52	40	45
Some post-high school	43	30	43	36	43	33
Baccalaureate or more	25	33	11	11	17	22

**Smoking status, %**						

Current smoker	7	7	17	18	13	13
Not a current smoker	93	93	83	82	87	87

**Other**						

Multivitamins 6 or more days/week, %	37	72	25	44	30	57
Age, mean, y (SD)	47.6 (14.1)	54.1 (13.2)	43.1 (9.4)	45.2 (11.2)	45.1 (11.8)	49.4 (12.9)
Body mass index, mean kg/m2^2^ (SD)	28.0 (5.4)	29.2 (7.0)	26.9 (3.9)	27.2 (3.9)	27.4 (4.6)	28.2 (5.7)
Fruits and vegetable consumption, mean servings/day (SD)	3.0 (1.3)	3.8 (1.7)	3.2 (1.7)	3.8 (1.9)	3.1 (1.6)	3.8 (1.8)
Red meat consumption, mean servings/wk (SD)	4.7 (3.5)	4.0 (2.9)	4.8 (3.2)	5.5 (4.0)	4.8 (3.3)	4.8 (3.6)
Physical activity, mean h/wk (SD)	3.6 (3.4)	4.0 (3.7)	5.8 (4.8)	5.7 (4.5)	4.8 (4.4)	4.9 (4.2)
Multivitamin use, mean no. of tablets/wk (SD)	2.9 (3.3)	5.3 (2.9)	2.4 (3.0)	3.3 (3.4)	2.6 (3.1)	4.2 (3.3)

**Table 2:** Pooled Plasma Nutrient Concentrations in the Healthy Directions Studies, by Usual Care and Intervention Groups

**Nutrient**	**Pooled Plasma Nutrient Concentration, μg/L**		***t* Test*P* value**

	**Usual Care (n = 49 Pairs) Mean (SD)**	**Intervention (n = 58 Pairs) Mean (SD)**	
Retinol	530.5 (103.5)	587.3 (119.8)	.01
α-Carotene	47.0 (27.7)	64.0 (48.7)	.03
*cis-*β-Carotene	15.3 (8.1)	18.7 (10.9)	.07
*trans-*β-Carotene	179.2 (99.1)	222.4 (132.9)	.06
Total β-carotene	194.4 (107.1)	241.0 (143.9)	.06
*cis-*Lycopene	234.9 (81.0)	249.2 (75.3)	.35
*trans-*Lycopene	228.6 (84.1)	240.1 (66.2)	.44
Total lycopene	463.5 (162.2)	489.3 (138.8)	.38
Lutein and zeaxanthin	201.0 (67.8)	194.7 (64.5)	.63
β-Cryptoxanthin	80.3 (38.6)	80.0 (37.2)	.97
α-Tocopherol	12,302.0 (3,794.2)	13,612.0 (4,077.7)	.09
δ-Tocopherol	294.7 (102.9)	255.5 (58.1)	.02
γ-Tocopherol	2,470.3 (877.1)	1,941.9 (840.2)	.002

**Table 3:** Pooled Plasma Nutrient Concentrations in the Healthy Directions Studies, by Multivitamin Use

**Nutrient**	**Pooled Plasma Nutrient Concentration, μg/L**		***t* Test*P* Value**

	**Infrequent Multivitamin Use^a^ (n = 36 Pairs) Mean (SD)**	**Multivitamin 6 or More Days/Wk (n = 24 Pairs) Mean (SD)**	
Retinol	501.7 (86.7)	609.5 (111.2)	<.001
α-Carotene	54.4 (38.8)	50.3 (26.3)	.62
*cis*-β-Carotene	13.7 (6.8)	21.5 (13.0)	.01
*trans-*β-Carotene	165.8 (86.6)	250.9 (156.3)	.02
Total β-carotene	179.3 (93.4)	272.4 (169.1)	.02
*cis-*Lycopene	239.8 (77.9)	231.2 (80.3)	.68
*trans-*Lycopene	229.4 (75.9)	219.4 (69.6)	.60
Total lycopene	469.1 (150.8)	450.6 (147.3)	.64
Lutein and zeaxanthin	201.5 (66.7)	183.0 (39.3)	.18
β-Cryptoxanthin	78.3 (38.6)	82.9 (33.2)	.62
α-Tocopherol	10,967.3 (2,866.3)	16,235.5 (4,578.0)	<.001
δ-Tocopherol	291.6 (87.7)	253.9 (69.8)	.07
γ-Tocopherol	2,587.7 (842.5)	1,742.7 (1077.1)	.002

a Mean (SD) days/wk among infrequent users = 0.6 (1.4).

**Table 4:** Pooled Plasma Nutrient Concentrations in the Healthy Directions Studies, by Fruit and Vegetable Consumption

**Nutrient**	**Pooled Plasma Nutrient Concentration, μg/L**		***t* Test*P* Value**

	**Fewer Than 4 Servings/Day (n = 53 Pairs) Mean (SD)**	**4 or More Servings/Day (n = 13 Pairs) Mean (SD)**
Retinol	548.9 (104.5)	602.2 (162.2)	.28
α-Carotene	50.6 (36.0)	64.2 (68.2)	.50
*cis-*β-Carotene	15.3 (8.0)	17.3 (7.6)	.39
*trans-*β-Carotene	180.6 (102.0)	209.3 (105.0)	.39
Total β-carotene	195.7 (110.0)	226.7 (112.4)	.38
*cis-*Lycopene	245.2 (83.2)	242.7 (87.3)	.93
*trans-*Lycopene	240.4 (81.1)	227.0 (77.1)	.59
Total lycopene	485.6 (161.3)	469.7 (160.7)	.75
Lutein and zeaxanthin	184.4 (60.3)	230.8 (74.1)	.05
β-Cryptoxanthin	75.8 (38.6)	101.8 (39.4)	.05
α-Tocopherol	12,720.1 (4,275.6)	13,708.5 (4,536.4)	.49
δ-Tocopherol	282.6 (93.9)	302.0 (63.5)	.38
γ-Tocopherol	2,347.4 (944.4)	2,232.1 (764.3)	.65

**Table 5:** Correlation of Pooled Plasma Nutrient Concentrations With Pair-Averaged Reported Multivitamin Intake (0–7 Days/Wk) in the Healthy Directions Studies

**Nutrient**	**Pooled Plasma Nutrient Concentration Correlation With Multivitamin Intake**					

	**Usual Care Group (n = 49 Pairs)**		**Intervention Group (n = 58 Pairs)**		**Combined Groups (n = 107 Pairs)**	

	**Spearman Correlation Coefficient, *r***	***P* Value**	**Spearman Correlation Coefficient, *r***	***P* Value**	**Spearman Correlation Coefficient, *r***	***P* Value**
Retinol	0.26	.08	0.33	.01	0.36	<.001
α-Carotene	0.04	.77	−0.19	.15	0.02	.87
*cis-*β-Carotene	0.39	.01	0.21	.11	0.34	<.001
*trans-*β-Carotene	0.34	.02	0.14	.31	0.29	.002
Total β-carotene	0.33	.02	0.14	.29	0.29	.002
*cis-*Lycopene	0.00	.98	−0.23	.09	−0.07	.44
*trans-*Lycopene	0.03	.82	−0.22	.10	−0.06	.55
Total lycopene	0.01	.96	−0.22	.10	−0.07	.47
Lutein and zeaxanthin	−0.05	.76	−0.09	.48	−0.08	.43
β-Cryptoxanthin	0.07	.65	0.10	.46	0.09	.37
α-Tocopherol	0.35	.01	0.54	<.001	0.49	<.001
δ-Tocopherol	−0.20	.17	−0.19	.15	−0.24	.01
γ-Tocopherol	−0.38	.01	−0.40	.002	−0.44	<.001

**Table 6:** Correlation of Pooled Plasma Nutrient Concentrations With Pair-Averaged Reported Fruit and Vegetable Intake (0–9 Servings/Day) in the Healthy Directions Studies

**Nutrient**	**Pooled Plasma Nutrient Concentration Correlation With Fruit and Vegetable Intake**					

	**Usual Care Group (n = 49 Pairs)**		**Intervention Group (n = 58 Pairs)**		**Combined Groups (n = 107 Pairs)**	

	**Spearman Correlation Coefficient, *r***	***P* Value**	**Spearman Correlation Coefficient, *r***	***P* Value**	**Spearman Correlation Coefficient, *r***	***P* Value**
Retinol	0.02	.88	0.02	.86	0.06	.52
α-Carotene	0.24	.10	0.10	.44	0.22	.02
*cis-*β-Carotene	0.34	.02	0.17	.21	0.28	.003
*trans-*β-Carotene	0.39	.006	0.15	.27	0.29	.002
Total β-carotene	0.39	.006	0.14	.28	0.29	.003
*cis-*Lycopene	0.15	.31	0.02	.85	0.09	.38
*trans-*Lycopene	0.15	.30	−0.01	.92	0.06	.55
Total lycopene	0.14	.33	0.01	.93	0.07	.45
Lutein and zeaxanthin	0.31	.03	0.23	.09	0.24	.01
β-Cryptoxanthin	0.35	.01	0.23	.08	0.27	.005
α-Tocopherol	0.06	.67	0.06	.65	0.09	.33
δ-Tocopherol	−0.07	.62	0.18	.17	0.02	.85
γ-Tocopherol	−0.16	.28	−0.03	.84	−0.16	.10
